# The Relationships between Effortful Control, Mind Wandering, and Mobile Phone Addiction Based on Network Analysis

**DOI:** 10.3390/healthcare12020140

**Published:** 2024-01-08

**Authors:** Rui Qiu, Zhihua Guo, Xianyang Wang, Xinlu Wang, Sizhe Cheng, Xia Zhu

**Affiliations:** Department of Military Medical Psychology, Air Force Medical University, Xi’an 710032, China; qiurui0417@163.com (R.Q.); zhguo1016@163.com (Z.G.); wangxianyang_1999@163.com (X.W.); wxl961010@163.com (X.W.); zhesicheng@163.com (S.C.)

**Keywords:** effortful control, mind wandering, mobile phone addiction, network analysis

## Abstract

Background: The prevailing mobile phone use brought the problem of addiction, which might cause negative consequences. Effortful control and mind wandering were associated with addictive behavior. The present study aimed to investigate the dimension-level relationships between effortful control, mind wandering, and mobile phone addiction. Methods: A total of 1684 participants participated this study. The mobile phone addiction, effortful control, and mind wandering were measured through self-report scales, respectively. Dimension-level network of these psychological variables was estimated and bridge expected influence (BEI) values for each node was calculated. Results: Dimensions of mobile phone addiction, effortful control, and mind wandering exhibited distinct and complex links to each other. The node “activation control” exhibited the highest negative BEI value (BEI = −0.32), whereas “spontaneous thinking” showed the highest positive BEI value (BEI = 0.20). Conclusions: Different dimensions of effortful control and mind wandering had varied yet significant connections with distinct dimensions of mobile phone addiction, facilitating understanding of the specific pathways underlying the three constructs. The identified dominant bridge nodes can provide potential targets for the intervention of mobile phone addiction.

## 1. Introduction

In our modern intelligent era, mobile phones play a key role in everyday life, integrating functions such as communication, entertainment, education, and work [1,2,3]. Studies have indicated that the number of Chinese netizens is growing, with 99.8% of them using mobile phones to access the Internet [4]. As a result, the risk of mobile phone addiction is on the rise [5,6]. Research has shown that mobile phone addiction can lead to various physical, mental, and social problems [7]. For example, Lemola et al. found that the duration of mobile phone use was negatively correlated with sleep time and positively correlated with dyscoimesis [8]. Darcin et al. discovered that mobile phone addiction could lead to social anxiety and higher levels of loneliness [9]. Elhai reported that mobile phone overuse during the COVID-19 pandemic could result in negative emotions such as anxiety and depression [10]. These cognitive, emotional, and behavioral issues caused by mobile phone addiction can have potential negative impacts on both individuals and society at large [11]. Therefore, scholars are currently intent on exploring the influencing factors for mobile phone addiction and ways to reduce its incidence.

Many studies have examined the risk factors associated with mobile phone addiction. For example, neurotic personality has been reported to be positively associated with a tendency toward mobile phone addiction [12]. Moreover, loneliness has been linked to higher mobile phone usage frequency [13]; while negative coping styles were positively correlated with mobile phone addiction scores [14]. In their exploration of risk factors, researchers have put forward several theoretical models. For example, the Interaction of Person-Affect-Cognition-Execution model proposed by Brand et al. in 2016 suggested that executive function was related to addiction susceptibility [15]. Billieux and others put forward the antisocial approach in the comprehensive model in 2015, claiming that emotional and impulsive personality traits were risk factors for problematic mobile phone use [16]. Lambert and others put forward the result expectation model in 2011, which held that individual’s cognitive function played an important role in the formation of mobile phone addiction [17]. These models generally tended to focus on effortful control, a dimension of individuals’ executive function. Effortful control refers to the ability to restrain automatic behavior and adhere to one’s target [18]. This ability comprises three dimensions: activation control, inhibitory control, and attentional control [19,20,21,22,23,24,25]. Individuals with a higher level of effortful control can better concentrate, divert their attention, and restrain inappropriate reactions in a timely fashion, particularly in situations involving avoidance tendencies [26]. Smartphone addicts tend to pay attention to network-related clues, which leads to attention difficulties [27,28]. In the early stage of the inhibition process, smartphone addicts will have more contradictions and obvious inhibition function defects, and this defect which is not limited by mobile phone-related stimuli [29]. Individuals who are addicted to or overuse smart phones are worse at implementing control system and impulsiveness, self-reported problematic smartphone usage was associated with deficits in latent factor task-switching [30,31]. Impulsivity is an important personality trait associated with mental health problem [32]. Impulse is often referred to as risk-taking choices, lack of planning, the tendency to act prematurely, poor ability to inhibit priming responses, and non-reflective choices for immediate rewarding response [33,34]. Impulse often leads to numerous negative consequences, including interpersonal relationships, social issues and criminal behavior [35]. Numerous studies have shown that effortful control ability acts as a protective factor with regard to negative events and adaptation issues [36,37]. Individuals with greater effortful control ability are better able to adapt to their environment, delay gratification, and control their unreasonable dependence and desire on mobile phones [38,39]. Based on the path model of mobile phone addiction proposed by Billieux et al. (2015), it is also known that the individual will control ability is one of the important factors affecting mobile phone addiction [40,41]. Based on the antecedent model of Internet addiction, it can be seen that there is a significant correlation between the level of addiction and the will control ability, and that the low ability of will control is considered as a significant risk factor in the occurrence and development of addiction [42,43]. Several studies have yielded results in support of this perspective [44,45]. Meanwhile, mind wandering is a phenomenon in which an individual loses their focus on the current moment and shifts their attention from the current task to unrelated information [46]. The information flow of smart phones is endless, and a vast amount of information can be obtained at any time and place. The user’s dependence on mobile social media and the frequency of use increase sharply, resulting in the fear of missing out and the phenomenon of mind wandering [47].This phenomenon increases the baseline of thinking output and induces addictive behavior of frequent or habitual examination [48]. Individuals with a higher level of mind wandering have more concerns in their lives, are more prone to information overload, and rely more on smart phones as an information source, which disrupts daily life and task performance [49]. At the same time, smartphone addicts will use mobile phones more in their daily lives, and the mobile phone itself, as well as the information it receives will interfere with the individual’s concentration state, forming a negative addiction loop [50]. Mind wandering can be divided into three dimensions: spontaneous thinking, attention out of control, and overall evaluation [51]. As a typical representative of today’s media, mobile phones are the most easily accessible distraction for contemporary individuals. Seizing human cognitive resources will lead to distraction and cognitive failure. Individuals who are more addicted to mobile phones will have more cognitive impairment. As described by the control failure and concern theory, mind wandering is closely intertwined with execution control ability [51]. Studies have shown that when mind wandering occurs, task performance tends to decline, and individuals experience negative emotions more frequently [52,53], leading to self-blame, self-denial, and even self-abandonment [54]. As a result, individuals become addicted to the virtual world created by mobile phones, seeking pathological compensation to satisfy their needs [55,56,57,58]. Although cell phone addiction is not included in DSM-5 or ICD-11, the above studies support that … (MPA) is a real addictive addiction disorder. Mobile phone addiction refers to physical, psychological and social function problems caused by indulging in mobile phone use phones [59].

In recent years, the network analysis model has been widely used in psychopathology, offering a new perspective for understanding a range of psychiatric disorders [60,61,62,63,64]. It also provides a fresh direction for identifying intervention and therapeutic targets for mental illnesses. By employing the network analysis model, we can better comprehend the interconnections between the dimensions of effortful control, mind wandering, and mobile phone addiction, leading to a deeper understanding of the pathological pathways involved.

In the network analysis method, two characteristics, namely the local quality of the network and the overall network structure, are utilized to investigate network characteristics [65]. A network analysis model consists of nodes and edges. Nodes represent different symptom dimensions in the network, while edges between nodes indicate partial correlations between dimensions. Different types of edges represent various relationships, including the connectivity and similarity between nodes. The bridge centrality index, for example, assesses the sum of edge weights between a node and nodes in other communities, helping to identify bridge nodes and to evaluate the interrelated nodes’ functions within the network. In doing so, these indices assist in identifying effective targets for prevention and intervention [66]. In addition, the network connectivity, as co-constructed from overall strength and network density, reflects the overall closeness of the network connections. Greater connectivity leads to faster diffusion and activation, and increased sensitivity to potential obstacles [67]. By applying the above theories and methods, we can analyze the psychopathological mechanisms of mobile phone addiction from a new perspective.

Based on network pathology, various symptom dimensions may exert different influences on individuals’ tendencies toward mobile phone addiction [68,69]. However, studies to date have often treated effortful control and mind wandering frequency as undivided wholes, evaluating individuals’ mental states by calculating total scores across all items. Unfortunately, the scholars who conducted these studies did not thoroughly investigate and analyze the relationships between these variables at the level of dimensions, thereby overlooking the heterogeneity and importance of the different dimensions [70,71]. To fill this gap, this study incorporates effortful control mind wandering into the analysis framework, collecting cross-sectional data to establish a network model for mobile phone addiction and its influencing factors. This approach offers new insights into mobile phone addiction from a finer-grained perspective. This study also assumes that the different dimensions of effortful control mind wandering have distinct connections with symptoms of mobile phone addiction, in that specific dimensions are expected to serve as key factors influencing specific symptoms of mobile phone addiction.

## 2. Methods

### 2.1. Study Design and Participants

This study was conducted in the form of online survey through Wenjuanxing platform (https://www.wjx.cn/, accessed on 1 March 2023) from 1 March 2023 to 23 March 2023. A total of 1932 healthy adults aged 18–54 years were recruited in the study by convenience sampling. Participants expressed their informed consent and were told that they could quit the study at any time. In the first part of the survey, we emphasized the anonymity of the research to encourage honest answers. In this research, we estimate the filling time of the questionnaire from three aspects: general experience, the number of questions in the questionnaire and the format of the questions in the questionnaire. At the same time, in SPSS 26.0 descriptive statistics are made on the reaction time, histograms are drawn, and the reaction time is quantitatively checked, and finally the screening range is determined. After excluding those who provided repetitive answers or who had excessively long or short response times, 1684 valid questionnaires were collected, with an effective response rate of 87.16%. Among participants, 82.13% were males; 32.42% were children; 21.6% were 20 years old and below; 57.4% were between 21–30 years old; 12.5% were between 31–40 years old; 4.5% were between 41–50 years old; and 4.0% were 51 years old and above. In terms of educational background, 3.0% had junior high school diplomas and below; 52.2% had senior high school and junior college diplomas; 43.6% had college degrees; and 1.2% had postgraduate qualifications and above.

### 2.2. Measures

#### 2.2.1. Mobile Phone Addiction Tendency Scale

This study used the Mobile Phone Addiction Tendency Scale (MPATS) developed by Xiong et al. [72] to measure mobile phone addiction. The reliability coefficient of the original scale Cronbach’s α is 0.83. The scale consists of 16 items scored on a 5-point Likert scale (ranging from 1 = strongly disagree to 5 = strongly agree) [72]. The total possible scores on this scale range from 16 to 80, measuring factors including withdrawal symptoms, salience, social comfort, and mood changes. Each subscale’s scores were calculated and summed up. The higher the total scores, the stronger the individual’s tendency toward mobile phone addiction. Withdrawal symptoms refer to negative physiological or psychological reactions when not participating in mobile phone activities (e.g., item “I will feel uneasy if I’m away from the phone for a long time”); salience refers to the centrality of mobile phone use in thinking and behavior activities (e.g., item “I often focus on the phone, which affects my lessons or work”); social comfort refers to the role of mobile phone use in interpersonal communication (e.g., item “I feel more comfortable when I communicate with others on the phone”); mood changes refer to the emotional changes caused by mobile phones (e.g., item “I will feel anxious and even lose my temper when my phone cannot connect or receive signals”). In this study, the Cronbach’s α values was 0.934 for the overall scale. The structural validity is *χ*^2^/*df* = 1325.486/98, RMSEA = 0.086, CFI = 0.929, TLI = 0.913, SRMR = 0.036.

#### 2.2.2. Effortful Control Questionnaire

The Effortful Control Questionnaire developed by Ellis et al. [73] and revised by Li et al. [74] was used for this study. The reliability coefficient of the original scale Cronbach’s α is 0.76. After revision and measurement, the scale comprised 15 items scored on a 5-point Likert scale (ranging from 1 = strongly disagree to 5 = strongly agree) [73,74]. The total possible scores on this scale range from 15 to 75 and the scale encompasses three factors: activation control, inhibitory control, and attentional control. Higher scores indicate stronger effortful control. Activation control involves performing an action when there is a strong tendency to avoid it (e.g., item “When I have something difficult to do, I will attempt it at once”); inhibitory control involves suppressing inappropriate responses (e.g., item “It’s easy for me to concentrate on finishing the task”); and attentional control involves better focus and attention shifting (e.g., item “I am a good observer of various different things happening around me”) [19,20,21,22,23,24,25]. The Cronbach’s α value for this scale was 0.707 in the current study. The structural validity is *χ*^2^/*df* = 4272.721/87, RMSEA = 0.169, CFI = 0.623, TLI = 0.546, SRMR = 0.164.

#### 2.2.3. Mind Wandering Questionnaire

The Mind Wandering Questionnaire developed by Song et al. (2011) was adopted for this study. The reliability coefficient of the original scale Cronbach’s α is 0.88. The scale comprises 22 items, divided into three dimensions: spontaneous thinking means the frequency at which scattered thoughts or imagination appear in mind (e.g., item “I will be interrupted by some ideas that appear in my mind”); attention disorder means the extent to which difficulty of maintaining a focus on your own attention (e.g., item “When I was reading a book, I noticed that I was not thinking, so I had to return and read it again”); overall evaluation means the degree to which ideologies are in a free state and beyond your own control (e.g., item “I feel that there is always a void in my mind”) All items are scored on a 5-point Likert scale (ranging from 1 = hardly to 5 = always) [75,76]. The total possible scores on this scale range from 22 to 110 and higher scores indicate a higher tendency toward mind wandering. In the current study, the Cronbach’s α value for this scale was 0.97. The structural validity is *χ*^2^/*df* = 1312.814/186, RMSEA = 0.060, CFI = 0.931, TLI = 0.923, SRMR = 0.033.

### 2.3. Statistical Analysis

SPSS 26.0 software was used for the descriptive analysis. The network structure was constructed using the RStudio (version 4.1.1) [77,78]. The validity of the scale was analyzed by using Mplus 8.3 software, including structural validity, aggregate validity and differential validity. Weight and bridge centrality evaluation of each node were calculated as well [79].

#### 2.3.1. Network Structure

We used qgraph package in R to construct the network [80,81]. The network consisted of nodes and edges. Each node represented one item in the scale, and between two nodes were edges representing the strength of connection in between. Each node was colored based on the psychological variable of each scale, forming three different communities in the network. Each edge was colored based on the positive or negative correlation it represented, and the thicker the edges the stronger the correlation [82]. The least absolute shrinkage and selection operator (LASSO) regularization and the Extended Bayesian Information Criterion (EBIC) were used to limit the emergence of spurious edges [83,84].

According to the recommended methods to investigate the accuracy of network inference, we routinely evaluated the stability of the network using the bootnet package. We calculated the Confidence Interval (CI) of each edge by non-parametric bootstrapping to examine to which extent the edge weight might have differed with one another [85]. The narrower the 95%CI, the more accurate edge weight and the more reliable the network.

#### 2.3.2. Bridge Centrality Evaluation

We used the networktools package to evaluate the bridge centrality of each node [86]. In this study, we calculated bridge expected influence (BEI) values of each node. The BEI value was defined as the total edge weights one node connected with other nodes outsides its own community. The higher the BEI value, the closer it correlated with other communities [87].

The bootnet package was used for the stability test and the difference test of BEIs. We conducted case-dropping bootstrapping to test the stability of BEI. The correlation stability (CS) coefficient was applied to quantify the stability, and a CS coefficient exceeding 0.25 indicated an acceptable stability [82]. Moreover, we conducted bootstrapping to test the differences of the BEI indices of different nodes as well.

## 3. Results

### 3.1. Descriptive Statistics

Table 1 shows the abbreviations, mean scores, standard deviations, and BEI values of each dimension of mobile phone addiction, effortful control, and mind wandering.

### 3.2. Network Analysis

**Figure 1:**
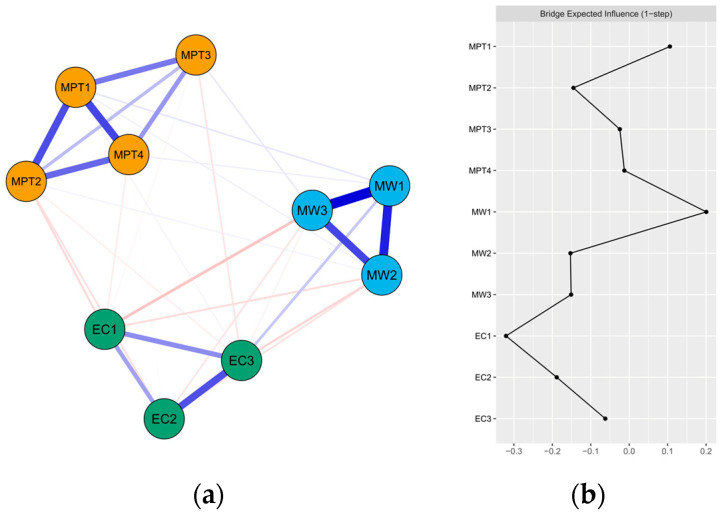
Network structure and raw values of bridge expected influence for each node in the present network. (**a**) EBICglasso network, where blue edges represent positive correlations and red edges represent negative correlations. A thicker and more saturated edge reflects a higher correlation between nodes; (Yellow circle: mobile phone addiction community, Green circle: Effortful control, blue circle: Mind Wandering) (Withdrawal symptoms: MPT1; Salience: MPT2; Social comfort: MPT3; Mood changes: MPT4; Activation control: EC1; Inhibitory control: EC2; Attentional control: EC3; Spontaneous thinking: MW1; Attention out of control: MW2; Overall evaluation: MW3) (**b**) Centrality plot depicting the BEI of each node in the network (raw value). The specific meanings of each node are shown in Table 1. The horizontal axis represents the BEI value and the vertical axis represents the node name.

Figure 1a shows the final network of mobile phone addiction, effortful control, and mind wandering, which comprises 10 nodes. There were 34 edges with edge weights ranging from −0.12 to 0.51, including 22 cross-community edges. Of the cross-community edges, relatively important edges were identified. Among the associations with mobile phone addiction, MW1 “spontaneous thinking” was positively connected to MPT1 “withdrawal symptoms” (weight = 0.05). EC1 “activation control” and EC2 “inhibitory control” were negatively associated with MPT2 “salience” (weight = −0.08 and −0.06, respectively). EC3 “attentional control” was negatively linked to MPT3 “social comfort” (weight = −0.05). Additionally, some dimensions of effortful control and mind wandering were correlated. EC1 “activation control”, EC2 “inhibitory control”, and EC3 “attentional control” were all negatively associated with MW2 “attention out of control” (weight = −0.07, −0.06, and −0.08, respectively). EC1 “activation control” and MW3 “overall evaluation” were also negatively connected (weight = −0.12). All edge weights within the present network can be seen in Supplementary Material. The bootstrapped 95% CI was narrow (see Figure S1 in the Supplementary Material), suggesting that the edge weight estimation was accurate and reliable. The bootstrapped difference test for edge weights is shown in Figure S2 in the Supplementary Material.

Figure 1b presents the raw BEI values for each variable node within this network. EC1 “activation control” exhibited the highest negative BEI value (BEI = −0.32), whereas MW1 “spontaneous thinking” had the highest positive BEI value (BEI = 0.20). The CS coefficient of node BEI was 0.75, exceeding the preferably recommended threshold of 0.5, indicating that the estimation of BEI was adequately stable (see Figure S3 in the Supplementary Material). The results of bootstrapped difference test for node BEI are provided in Figure S4 in the Supplementary Material.

## 4. Discussion

To the extent of our knowledge, this is the first study to investigate the dimension-level network structure of mobile phone addiction, effortful control, and mind wandering using network analysis. The results demonstrated that there were relatively important pairwise relationships, i.e., cross-community edges, between the distinct dimensions of the three constructs. The present study also identified some dimensions as bridge nodes that played important roles in facilitating adverse or positive impacts on mobile phone addiction. Since no relevant research has been conducted yet, our results of this exploratory study are preliminary. However, these findings may advance our understanding of the specific pathways underlying mobile phone addiction, effortful control, and mind wandering, suggesting potential prevention and intervention targets against mobile phone addiction.

Notably, certain important cross-community edges were identified in the present network. MW1 “spontaneous thinking” was positively associated with MPT1 “withdrawal symptoms”. This reciprocal link accorded with previous studies revealing that mobile phone addiction and mind wandering were positively connected and that mobile phone addiction explained about one third of variance in their level of mind-wandering among university students [88,89]. Our finding further uncovered that the fine-grained interrelation between mind wandering, and mobile phone addiction can be attributable mainly to the specific connection between the spontaneous thinking and withdrawal symptoms.

Conversely, it should be noted that EC1 “activation control” and EC2 “inhibitory control” were negatively correlated with MPT2 “salience”; EC3 “attentional control” was negatively associated with MPT3 “social comfort”. These results were consistent with a published study showing that effortful control, as a protective factor, buffered the detrimental effect from certain variables to mobile phone addiction [44]. Another study also reported that effortful control fully mediated the effect of personality, such as conscientiousness, on mobile phone addiction [90]. In the refined exploration of the pathways between effortful control and mobile phone addiction, we found inhibitory control was associated with salience. Numerous literature have proposed that inhibitory control was closely related to addictive behaviors [42,91,92,93,94,95], and salience was an important constituent of addictive behaviors according to the addiction components model [43,96,97]. Hence, this association is reasonably understandable.

Regarding activation control and salience, activation control refers to perform an action when there is a strong tendency to avoid it and reflect the ability to plan ahead and act pro-socially [74,98]; activation control and inhibitory inhibition are identified as two subcomponents of self-regulatory capacity [19,98,99], which is relevant to dual process models of addiction in that addictive behavior is influenced by the interactive effect of the impulsive (reflexive/automatic/spontaneous) and self-regulatory (reflective/controlled/deliberative) processes [98,100,101,102]. Therefore, the negative link between activation control and salience component of mobile phone addiction was foreseeable.

Additionally, we also found attentional control was negatively associated with social comfort of mobile phone addiction. This finding was similar to a study showing that high level of attentional control was revealed to protect against addiction-related negative consequences [99]. Furthermore, self-regulation was often operationalized as effortful control and its facets (attentional, inhibitory, and activation control) [19,36,99,103]. Accordingly, attentional control—subdomain of self-regulation—can engage in preventing addiction according to the proposed dual-process model [98], and the underlying mechanism of action may lie in the negative connection between attentional control and social comfort.

In addition to associations with mobile phone addiction, we found certain negative connections between EC1 “activation control”, EC2 “inhibitory control”, EC3 “attentional control” and MW2 “attention out of control”, as well as between EC1 “activation control” and MW3 “overall evaluation”. These close relationships between dimensions of effortful control and mind wandering highlighted their ability to interactively exert indirect influence on mobile phone addiction via their respective pathways, indicating they are common factors affecting mobile phone addiction. These findings were in accordance with a published study that low effortful control indicated more mind wandering and effortful control acted as one of the key mechanisms underlying the functional influence of mind wandering on real-life outcomes [104]. Effortful control has been reported to be closely related to inattention of attention-deficit hyperactivity disorder [105,106], similarly accounting for the relations between attention out of control and three facets in effortful control. As for insights into the relation between activation control and overall evaluation, there are, to the best of our knowledge, no previous studies with which to compare our results. Our results extend previous study findings by providing a more nuanced perspective of the relations among the dimensions.

Based on the BEI values, EC1 “activation control” and MW1 “spontaneous thinking” were determined as important bridge nodes, indicating their critical roles in transmitting impacts among communities. EC1 “activation control” had a negative BEI, suggesting it acted as a protective factor against developing mobile phone addiction and experiencing mind wandering, whereas MW1 “spontaneous thinking” featured a positive BEI, indicating its facilitation leading to the development and maintenance of mobile phone addiction. These findings were in line with previous studies that effortful control was a protective factor, and, in contrast, mind wandering was a risk factor for mobile phone addiction [44,88,89,107]. As mentioned above, the three bridge nodes exhibited elaborate connections with dimensions of mobile phone addiction. Accordingly, this study provides further lines of evidence supporting relevant conclusions from the perspective of network theory.

The present study has two aspects of significant implication. Regarding the theoretical implication, this study examined the dimension-level interrelations between mobile phone addiction, effortful control, and mind wandering using network analysis, such as the edge between inhibitory control and salience. We believe that viewing data through this lens allows for a more granular understanding of the specific pathways underlying the three constructs. In other words, these findings are of importance to figure out specific roles (protective/detrimental) played by different dimensions of effortful control or mind wandering in the development and maintenance of dimensions of mobile phone addiction. Regarding practical implication, bridge nodes are crucial to the co-occurrence of psychological constructs and to facilitate the adverse or positive effects of one community on others [86]. Hence, bridge nodes are regarded as potential targets for prevention and intervention [86,108,109]. In this study, activation control and spontaneous thinking are identified as critical bridge nodes and hence are indicated as putative intervention targets, providing implications for clinical practice. For example, focusing on the protective role of activation control and preventing the negative effect of spontaneous thinking may increase the effectiveness of prevention and intervention against mobile phone addiction and benefit treatment outcomes.

## 5. Limitations

Notwithstanding the significance of the present study, several limitations existed. First, this study is a cross-sectional design and thus the causality between distinct dimensions cannot be inferred. The cross-sectional study employed in this study can study large samples at the same time, with strong representation of subjects, excellent generalization ability of results, strong timeliness, no repeated measurement problems and easy implementation. However, due to its lack of systematic continuity, it is difficult to determine the causal relationship between the occurrence and the development of mobile phone addiction. Therefore, in the following research, we will combine the longitudinal research method to test the psychological activities and characteristics of the subjects repeatedly in a specified period of time, so as to systematically and thoroughly understand the continuous process of the relationship between effective control, mind-wandering and mobile phone addition and the laws of quantitative change and qualitative change. Second, mobile phone addiction, effortful control, and mind wandering were measured using self-reported scales, which may be susceptible to subjective bias [110]. This implies that the results of our analysis should be interpreted with some caution. Third, although the findings suggested activation control and spontaneous thinking as potential intervention targets, it remains to be shown experimentally how big the actual effectiveness of the putative interventions will be. Fourth, the network structure constructed in this study reflects between-subject effects at a group level, indicating that it cannot capture idiographic individual-level processes. Finally, shortcomings also include limited generalizability of the results due to the data-driven nature of network analysis approach. The extension of our findings to other populations requires more validation.

## 6. Conclusions

The present study is the first to examine the relationships between mobile phone addiction, and effortful control and mind wandering using network analysis. The results demonstrated that there were significant correlations between the distinct dimensions of the three structures. Moreover, by analyzing their detailed relations, the problem of mobile phone addiction can be solved by enhancing effortful control and preventing mind wandering. Among them, the dimension of withdrawal symptoms has been identified as the key bridge node, which is closely linked with efficient control and distraction, and has become an effective indicator and a promising intervention target for identifying mobile phone addiction. This research provides a new perspective on the relationship between mobile phone addiction and effective control and distraction.

## Figures and Tables

**Table 1:** Abbreviations, mean scores, standard deviations, and BEI values for each study dimension.

Variables	Abb	M	SD	BEI
Mobile phone addiction				
Withdrawal symptoms	MPT1	2.39	0.87	0.11
Salience	MPT2	1.82	0.81	−0.15
Social comfort	MPT3	2.14	0.87	−0.02
Mood changes	MPT4	2.06	0.89	−0.01
Effortful control				
Activation control	EC1	3.68	0.67	−0.32
Inhibitory control	EC2	3.56	0.60	−0.19
Attentional control	EC3	3.35	0.49	−0.06
Mind Wandering				
Spontaneous thinking	MW1	2.00	0.85	0.20
Attention out of control	MW2	1.89	0.81	−0.15
Overall evaluation	MW3	1.85	0.81	−0.15

Abb, abbreviation; M, mean; SD, standard deviation; BEI, bridge expected influence.

## Data Availability

The datasets presented in this article are not readily available because the datasets involve unfinished research projects. If necessary, requests to access the datasets should contact the corresponding author.

## Supplementary Materials

The following supporting information can be downloaded at: https://www.mdpi.com/article/10.3390/healthcare12020140/s1, Figure S1: Accuracy of edge weights in the network of mobile phone addiction, effortful control, and mind wandering; Figure S2: Bootstrapped difference test for edge weights; Figure S3: Stability of node bridge expected influences in the network of mobile phone addiction, effortful control, and mind wandering; Figure S4: Bootstrapped difference test for node bridge expected influences in the present network.
