# Supplementary material for: The Relationships between Effortful Control, Mind Wandering, and Mobile Phone Addiction Based on Network Analysis
Source: Healthcare (Basel). 2024 Jan 8;12(2):140. doi: 10.3390/healthcare12020140 (PMC10815513; doi:10.3390/healthcare12020140)
Supplement: Supplementary file 1 [file healthcare-12-00140-s001.zip › healthcare-2790740-supplementary.pdf]

Supplementary Material

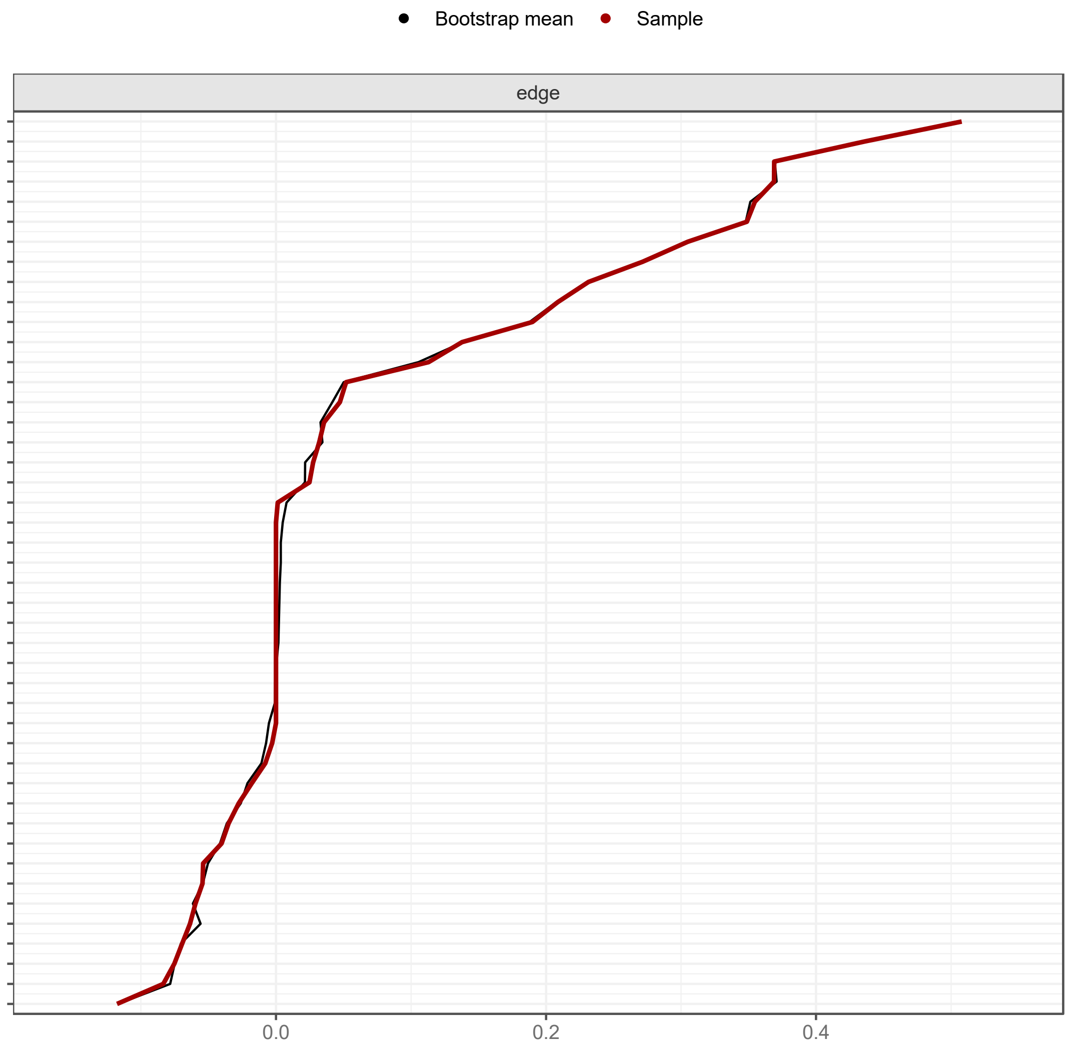

Figure S1. Accuracy of edge weights in the network of mobile phone addiction, effortful control, and mind wandering

Note: The red line depicts the sample edge weights, the black line represents bootstrap means, and the gray bar depicts the bootstrapped confidence interval.

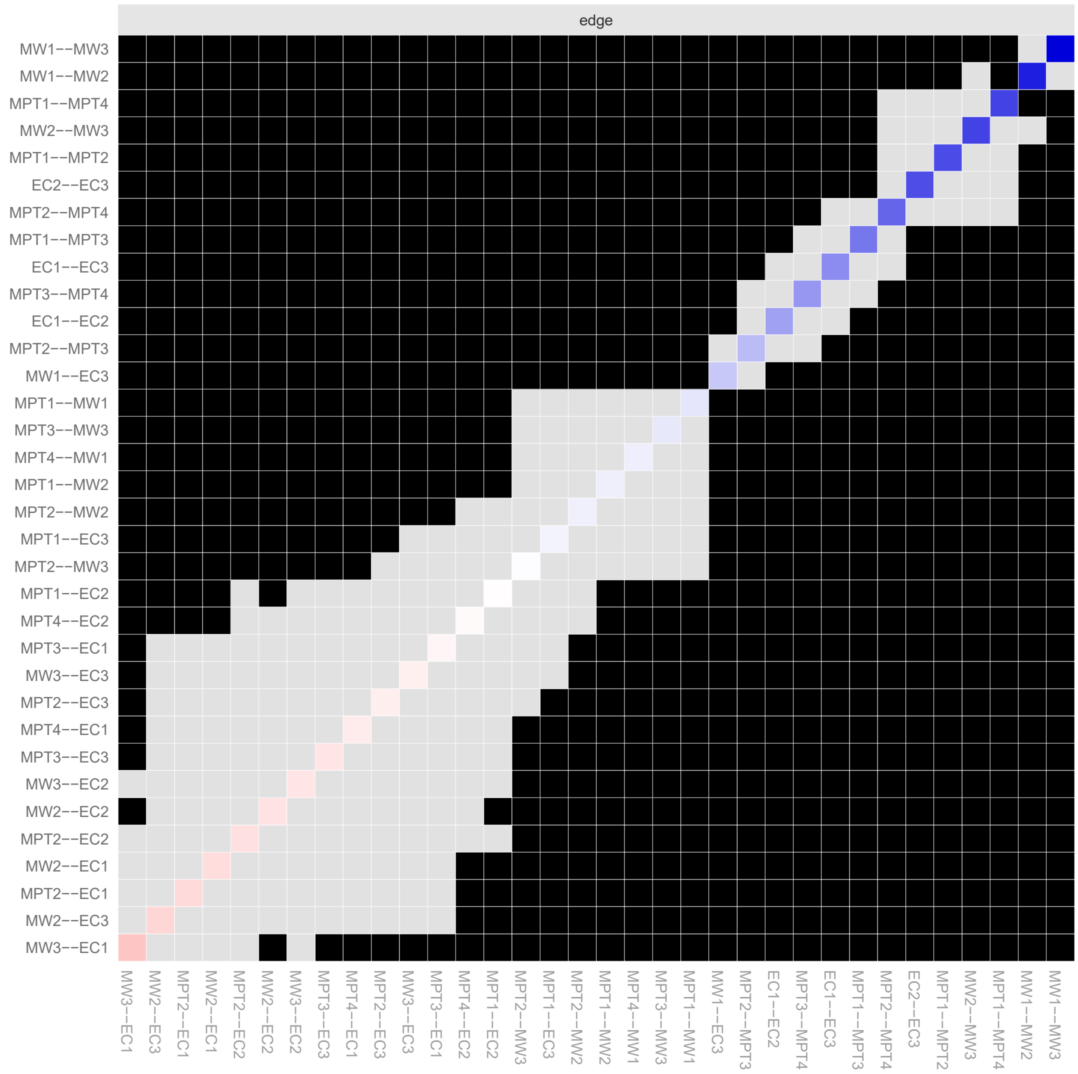

Figure S2. Bootstrapped difference test for edge weights

Note: Gray boxes indicate edge weights that do not differ significantly from one another, while black boxes indicate edge weights that do differ significantly. Blue and red boxes on the diagonal correspond to edge weights with positive and negative correlations, respectively.

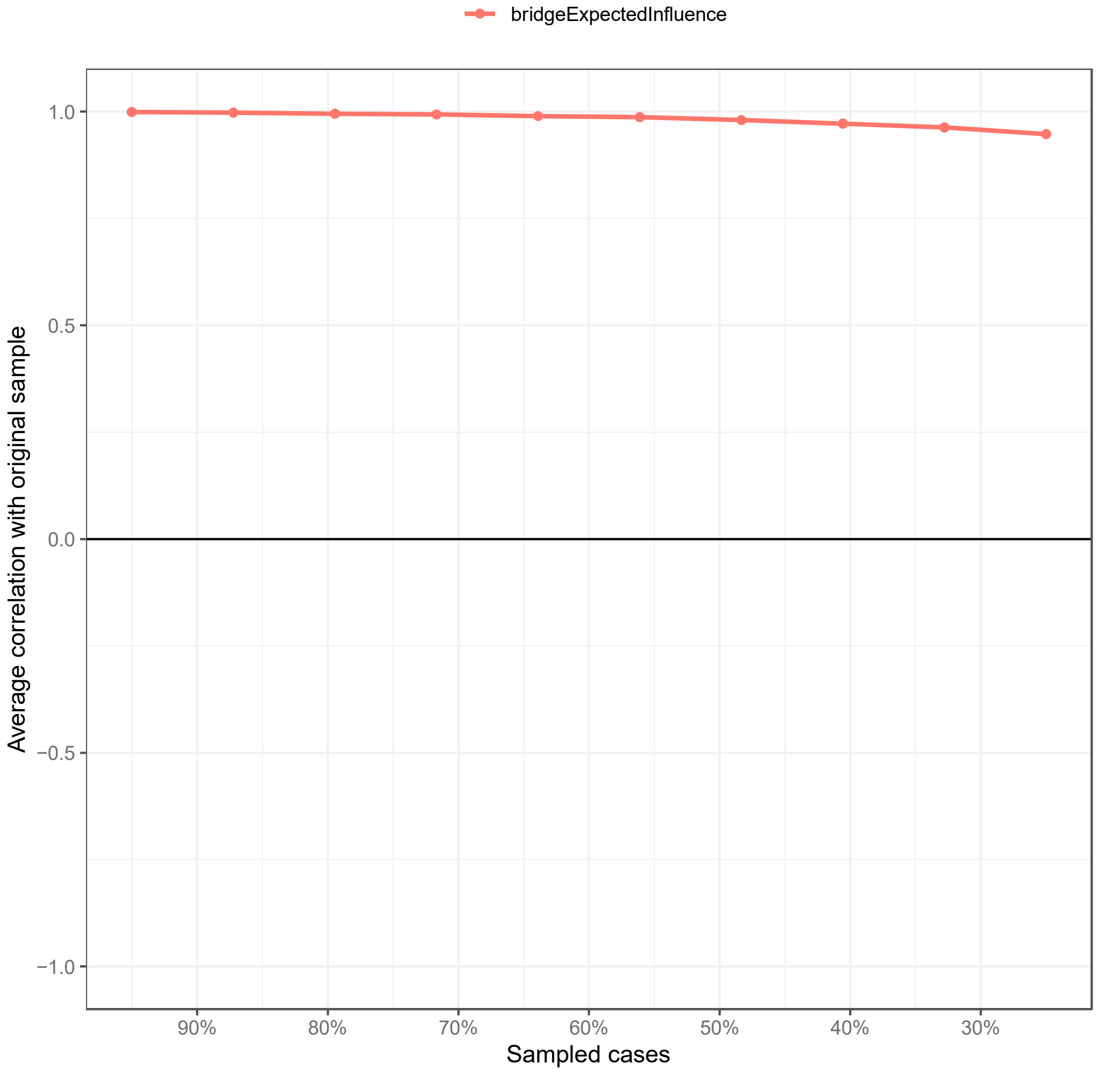

Figure S3. Stability of node bridge expected influences in the network of mobile phone addiction, effortful control, and mind wandering

Note: The red bar represents the average correlation between node bridge expected influences in the full sample and subsample with the red area depicting the 2.5th quantile to the 97.5th quantile.

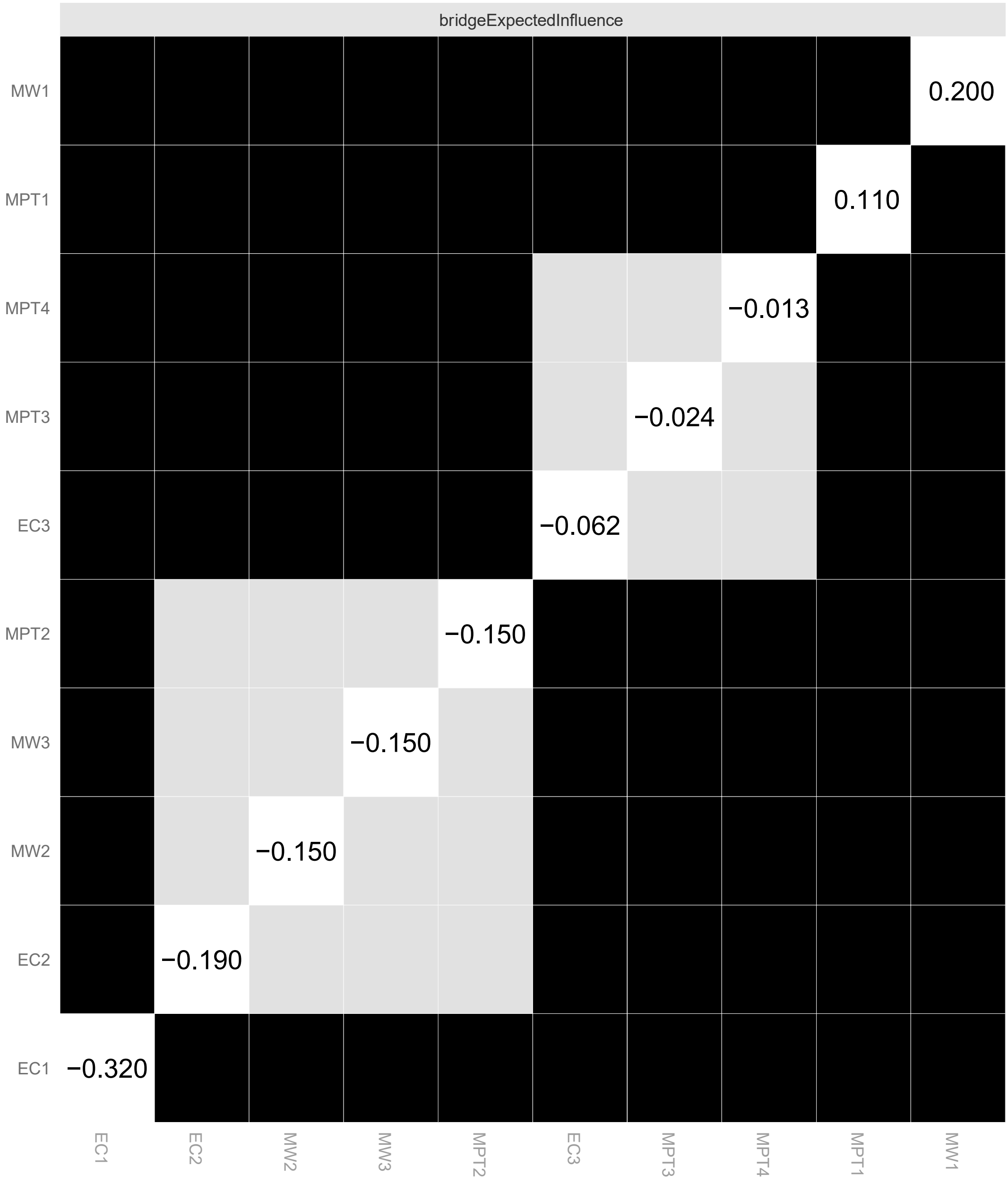

Figure S4. Bootstrapped difference test for node bridge expected influences in the present network

Note: Gray boxes indicate node bridge expected influences that do not differ significantly from one another, while black boxes indicate node bridge expected influences that do differ significantly. The number in the white boxes (i.e., diagonal line) represent the value of node bridge expected influences.
